# Poly(ADP-Ribose) Polymerase-3 Regulates Regeneration in Planarians

**DOI:** 10.3390/ijms21030875

**Published:** 2020-01-29

**Authors:** Paul G. Barghouth, Peter Karabinis, Andie Venegas, Néstor J. Oviedo

**Affiliations:** 1 Department of Molecular and Cell Biology, University of California, Merced, CA 95340, USA; pbarghouth@ucmerced.edu (P.G.B.); pkarabinis@ucmerced.edu (P.K.); avenegas25@ucmerced.edu (A.V.); 2 Quantitative and Systems Biology Graduate Program, University of California, Merced, CA 95340, USA; 3 Health Sciences Research Institute, University of California, Merced, CA 95340, USA

**Keywords:** PARP, planarians, stem cells, neoblasts, regeneration, apoptosis

## Abstract

Protein ADP-ribosylation is a reversible post-translational modification (PTM) process that plays fundamental roles in cell signaling. The covalent attachment of ADP ribose polymers is executed by PAR polymerases (PARP) and it is essential for chromatin organization, DNA repair, cell cycle, transcription, and replication, among other critical cellular events. The process of PARylation or polyADP-ribosylation is dynamic and takes place across many tissues undergoing renewal and repair, but the molecular mechanisms regulating this PTM remain mostly unknown. Here, we introduce the use of the planarian *Schmidtea mediterranea* as a tractable model to study PARylation in the complexity of the adult body that is under constant renewal and is capable of regenerating damaged tissues. We identified the evolutionary conservation of PARP signaling that is expressed in planarian stem cells and differentiated tissues. We also demonstrate that *Smed-PARP-3* homolog is required for proper regeneration of tissues in the anterior region of the animal. Furthermore, our results demonstrate, *Smed-PARP-3(RNAi)* disrupts the timely location of injury-induced cell death near the anterior facing wounds and also affects the regeneration of the central nervous system. Our work reveals novel roles for PARylation in large-scale regeneration and provides a simplified platform to investigate PARP signaling in the complexity of the adult body.

## 1. Introduction

Poly ADP-ribosylation (PARylation) is catalyzed by poly (ADP-ribose) polymerases (PARPs). The PARP family is evolutionarily conserved across different species and plays pivotal roles in the regulation of chromatin reorganization, DNA damage response, transcriptional regulation, apoptosis, and mitosis (e.g., PARP-1, 2, and 3) [1,2,3,4,5]. PARylation is an integral response that appears rapidly at sites of damaged DNA and establishes its effect through post-translational protein modifications [4,6,7]. Indeed, PARPs are well characterized for their activation by single and double-stranded DNA breaks through the use of NAD^+^ substrates [2,3,5,8,9]. Specifically, PARPs target proteins by the transfer of ADPR moieties through a 2’,1”-*O*-glycosidic ribose-ribose bond, thus producing a long and repetitive PAR chain containing an estimated 200 ADPR units in length [1,3,9]. However, the regulation and most of the PARP protein functions remain poorly understood.

Treatment with PARP inhibitors (PARPi) is a Food and Drug Administration (FDA) approved strategy aimed at self-renewing cancer cells that display high levels of PARP activation. In some cancers, the use of PARPi enhances the combined therapeutic efficacy of ionizing radiation, and they are considered a powerful tool against breast and ovarian cancer [10]. However, the mechanisms by which PARPi sensitize cancer stem cells and their potential side effects are still under investigation [10]. The role of PARylation has been equated to DNA damage-derived cell death as PARP activation leads to the cytosolic depletion of NAD^+^ and the release of mitochondrial apoptosis-inducing factor (AIF) [10]. Furthermore, maintenance of NAD^+^ and ATP is crucial for central nervous system longevity, and cognitive loss has been attributed to hyper-PARylation [11,12]. Thus, PARP proteins act as a mediator of neuronal death and a target for neuroprotection and neural regeneration [11,13,14,15]. Nonetheless, the presence of multiple PARP proteins along with their ubiquitous expression make it challenging to analyze their function in the adult body that is under constant repair and renewal of different tissues.

Planarian flatworms are members of the phylum Platyhelminthes with extraordinary regenerative capacity. The planarian *Schmidtea mediterranea* is widely used to study aspects of stem cell regulation during tissue renewal and regeneration [16,17]. Neoblasts are the planarian stem cells, which are constantly dividing to generate new cells required for cellular turnover of dozens of adult tissues (e.g., muscle, intestine, and nervous system). In the case of tissue injury, neoblasts divide, migrate, and their progeny differentiate to rebuild missing or damage tissues [16,17]. Recent work from our group has demonstrated planarians display high evolutionary conservation of DNA damage response and repair (DDR) signaling pathways during tissue homeostasis and regeneration [18,19,20]. Through in silico analysis of regenerating animals, it was determined that the planarian PARP homologue *Smed-PARP-3* was expressed independently from other DDR signaling genes during the generic wound response. However, the in vivo role of PARP signaling in neoblast regulation is unknown. Here, we identify three DNA-dependent PARP homologues and characterize their function during the process of tissue renewal and regeneration in *S. mediterranea*. Our results show that *Smed-PARP-3* signaling is critical for the proper regeneration of tissues in planarians. Specifically, we demonstrate disruption of *Smed-PARP-3* function alters cell death in anterior facing wounds, which is followed by reduced blastema size and dysfunctional regeneration of the nervous system. Altogether, our work introduces *S. mediterranea* as a tractable model system to explore the role of PARylation signaling during tissue renewal and regeneration in the complexity of the adult body.

## 2. Results

### 2.1. DNA Dependent PARylation is Evolutionarily Conserved in Schmidtea mediterranea

**Figure 1:**
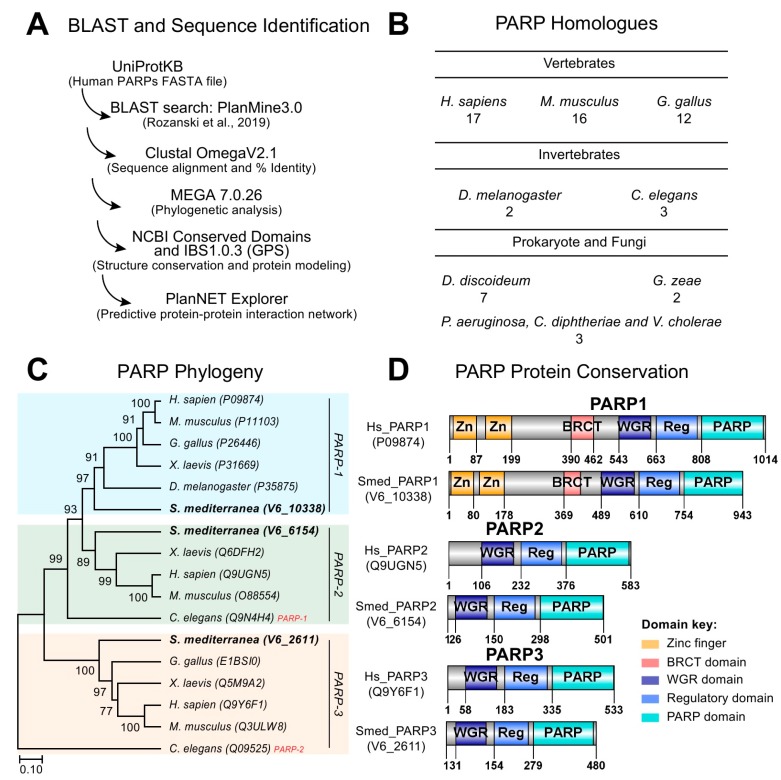
Conservation of DNA dependent PARylation signaling in planarian. (**A**) PARylation gene identification schematic and design. Human Query IDs were BLASTed into PlanMine 3.0 [24], resulting in a broad range of Smed ID hits outputs. (**B**) Number of PARP homologs across different species ranging from vertebrates and invertebrates to prokaryotes and fungi. (**C**) Phylogenetic bootstrap consensus tree of PARP-1, -2, and -3 (gene groupings are color coded, respectively) across an array of species using MEGA7 software. Analysis shows that planarian PARP homologues are clustered properly per PARP number unlike its close relative *C. elegans*. (**D**) Protein conservation modeling of Smed-PARP-1, -2, and -3 relative to the human counterpart. Signature domains of PARPs -1, -2, and -3 are the PARP domain, PARP regulatory domain (i.e., Reg.), and the tryptophan-glycine-arginine-rich (WGR) interacting domain. Key signatures of PARP-1 were found to be conserved in the planarian with BCRT and zinc finger (Zn) domains.

To identify whether PARP signaling is conserved in planarians, we used sequences corresponding to the 17 human PARP proteins and BLASTed them into the *S. mediterranea* genome (Figure 1A) [21]. Our search resulted in the identification of over 1600 Smed ID hits with many of these target sequences being redundant. Most of the hits consisted of partial domains, isolated signature domains, and/or completely lacking PARP-specific domains (e.g., Tankyrase, Macro, CCCH-, and PARP). Nonetheless, we were able to identify three bona fide human PARP homologs involved in DNA dependent functions. We called these DNA dependent PARP homologs *Smed-PARP-1*, *-2*, and *-3*, which is consistent with recent findings [20,22]. Future studies would be required to define the total number of PARP homologs in *S. mediterranea*. Thus, the initial characterization will only focus on PARP candidate sequences with putative DNA dependent functions (i.e., *Smed-PARP-1*, *-2*, and *-3*). The average number of conserved DNA dependent PARP homologs is similar to the ones found in other vertebrates, prokaryote, and fungi species (Figure 1B) [1,10,23].

DNA dependent PARP homologs, *Smed-PARP-1*, *-2,* and *-3* were highly conserved to the human counterparts with identities ranging from 41%, 61%, and 56%, respectively. We expanded the analysis by plotting the evolutionary relationships of taxa using the Bootstrap consensus tree and identified that all three *Smed-PARPs* cluster with their perspective PARP member across species (Figure 1C). Protein conservation for *Smed-PARP-1* included the signature PARP-1 zinc fingers and BRCT domains required for DNA-interaction. Moreover, all three homologues, *Smed-PARP-1*, *-2*, and *-3* contained the core WGR, PARP, and regulatory domains (Figure 1D). Altogether, our results suggest that members of PARP signaling involved in DNA-dependent functions appear evolutionarily conserved in *S. mediterranea*.

### 2.2. Smed-PARP Homologs are Mainly Expressed in Neoblasts and Post-Mitotic Cells

**Figure 2:**
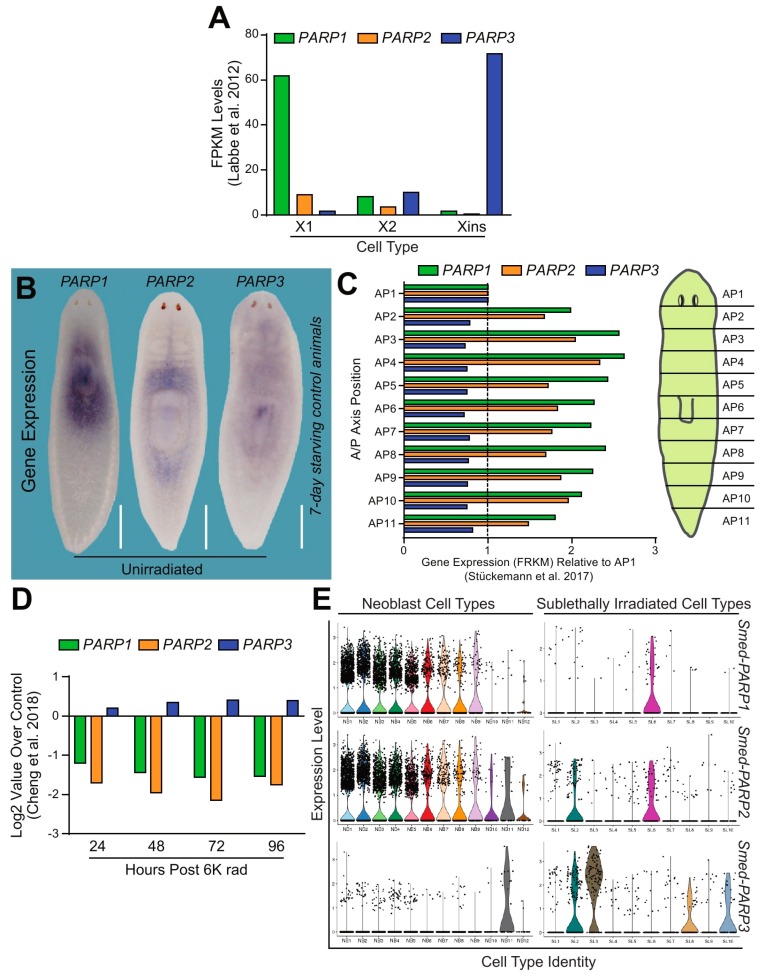
DNA dependent PARPs are highly expressed throughout the planarian. (**A**) Fragments per kilobase of exon model per million reads mapped (FPKM) levels depict gene expression of *Smed-PARP-1*, *-2*, and *-3* (i.e., green, orange, and blue, respectively). Data is derived from FACS-isolated single-cell RNA sequencing [27]. It is evident that *Smed-PARP-1* and *-2* are expressed in the neoblast and early progenitor populations (e.g., X1 and X2, reactively) while *Smed-PARP-3* is expressed within the differentiated (e.g., Xins) compartment. (**B**) Whole mount in situ hybridization probing for *Smed-PARP-1*, *-2*, and *-3* within 7-day starving control animals. Scale bar 200 µm. (**C**) Expression levels for *Smed-PARP-1*, *-2*, and *-3* across the 11 planarian anteroposterior axis quadrants derived from Stuckemann et al. [29]. Data represents the fold change in FPKM relative to the AP1 quadrant. (**D**) Expression levels during a 96-h time course post lethal (6000 rad) irradiation dose for *Smed-PARP-1*, *-2*, and *-3*. Data derived from Cheng et al. 2018 [31]. (**E**) Expression levels for *Smed-PARP-1*, *-2*, and *-3* determined from the Planosphere fate mapping atlas [35]. *Smed-PARP-1* and *-2* expression is widely distributed among the neoblast cell clusters and *Smed-PARP-3* within the neural neoblast cluster and the sub-lethally irradiated cell clusters of the nervous and pharyngeal tissues.

Next, we performed in silico analysis to learn about the gene expression distribution corresponding with *Smed-PARP-1*, *-2*, and *-3*. The analyses were performed using publicly available *S. mediterranea* genomic resources [21,24,25,26]. First, gene expression obtained from cells sorted with flow cytometry-FACS, revealed ubiquitous expression of all *Smed-PARP-1*, *-2*, and *-3* genes within neoblasts and post-mitotic progenitors (X1, X2, and Xins, respectively; Figure 2A) [27]. However, the expression of *Smed-PARP-1*, *-2*, and *-3* was not uniform across cell populations. Specifically, the expression levels of *Smed-PARP-1* were highly enriched within the X1 population, which include cells with >2n DNA (i.e., neoblasts in S/G2/M phases of the cell cycle), and the X2 cells that are thought to contain the immediate neoblast post-mitotic progeny and cells in G1 phase of the cell cycle [28]. *Smed-PARP-2* was also expressed mostly in X1 and X2 cells, albeit at lower levels than *Smed-PARP-1*. On the other hand, *Smed-PARP-3* expression was lower in X1 cells but highly enriched in the Xins, which includes post-mitotic and terminally differentiated cells (Figure 2A) [27].

To identify the spatiotemporal distribution of *Smed-PARP-1*, *-2*, and *-3*, we performed whole-mount in situ hybridization on 7-day starved animals. We identified that all three PARP genes were highly expressed in the area surrounding the pharynx and anterior tissues (Figure 2B). Furthermore, *Smed-PARP-1* expression is concentrated in the pharynx while *Smed-PARP-2* expression appears diffused around the pharyngeal area and the digestive system. The *Smed-PARP-3* expression pattern was broadly diffused throughout the animal (Figure 2B). Additional in silico analysis [29] evidenced a similar pattern of expression as the one found with in situ hybridization for the *Smed-PARP* genes (Figure 2C).

The expression of *Smed-PARP-1* and *-2* associated with neoblasts was confirmed by analyzing their transcription levels after exposure to lethal doses of ionizing radiation (i.e., 6000 rad) that is known to eliminate neoblasts and their immediate progeny irreversibly [27,30]. Upon irradiation *Smed-PARP-1* and *-2* expression was severely suppressed for over four days, while *Smed-PARP-3* transcription was still present (Figure 2D). Furthermore, we also observed similar results after the elimination of neoblasts by RNAi of crucial regulators such as *H2B*, *p53*, and *zfp-1* (Supplementary Figure S1A–C) [31,32,33]. Together, these results consistently show that PARP genes are differentially expressed across the planarian body with *Smed-PARP-1* and *-2* expression mostly associated with neoblasts, while *Smed-PARP-3*’s transcription is found within post-mitotic cells.

To discern the expression patterns of *Smed-PARP-1*, *-2*, and *-3* at the cellular level, we examined single-cell RNA sequencing using contig enrichment in the planarian Digiworm database [34] (Supplementary Figure S2A–C). We further confirmed that both *Smed-PARP-1* and *-2* were highly enriched within the neoblast main clusters (e.g., 0, 5, and 22) and the *smedwi+1* sub-cluster with expression levels seeming to highly overlap one another. On the other hand, *Smed-PARP-3* expression was highly expressed among differentiated cells, particularly in the neural clusters (Supplementary Figure S2C). Expanding the expression analysis to the recently created single-cell expression database Planosphere allowed us to obtain higher resolution about the type of neoblasts and neural cells transcribing *Smed-PARP* genes [35]. For example, the expression *Smed-PARP-1* and *-2* were enriched in the self-renewing neoblast compartments of the cNeoblast populations (i.e., NB2 and SL6) and their radiation-sensitive progenitor pools (i.e., NB1, NB3-9) with *Smed-PARP-2* expression expanding to NB10-12. *Smed-PARP-3* expression was high among the neural neoblast population (i.e., NB11) and sub-lethally irradiated neural, epidermal, and pharyngeal clusters (e.g., SL-2, -3, -8, and -10, respectively; Figure 2E and Supplementary Figure S3A,B). Altogether, the expression analyses incorporating independent in silico resources and the spatial distribution observed with in situ hybridization, demonstrate the expression of *Smed-PARP* genes is distributed among neoblasts and post-mitotic cells. The findings also indicate that the expression of the *Smed-PARP* genes is not homogenous across different cell types.

### 2.3. Smed-PARP Genes Regulate DNA Repair in Uninjured Animals

**Figure 3:**
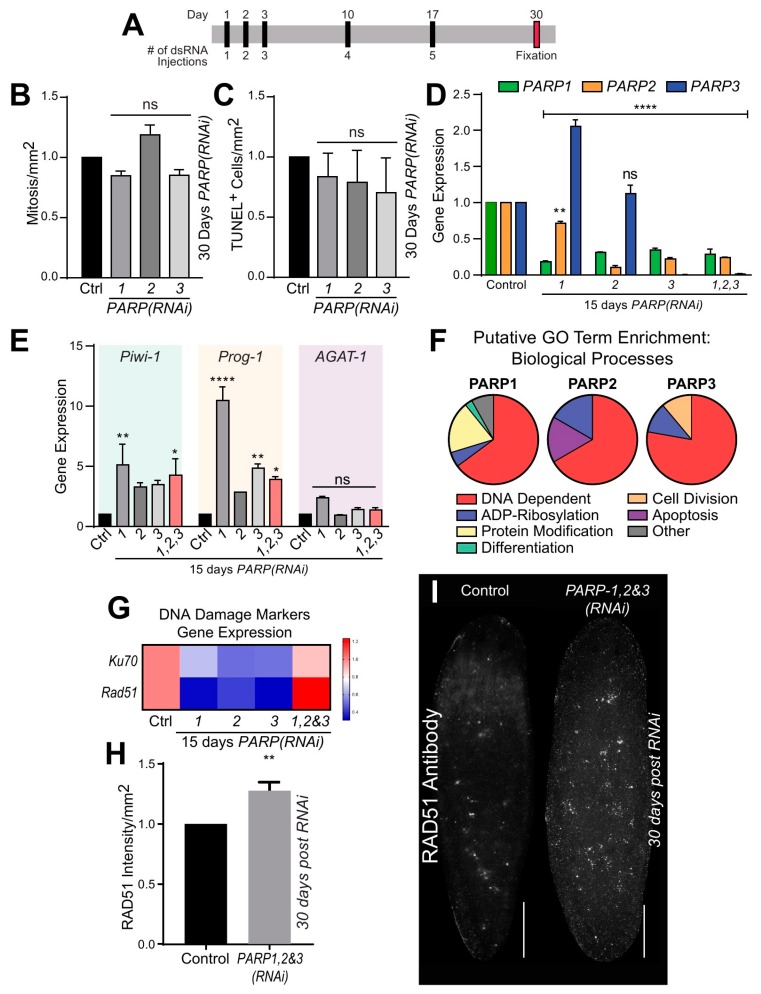
*Smed-PARPs* have a conserved role in the preservation of genomic stability during planarian cellular turnover. (**A**) Injection time course regimen consisting of five dsRNA microinjections throughout 30-days. (**B**,**C**) Quantification of mitotic events and cell death 30 dpfi, result in no significant alterations in events relative to the injected control. These results are derived from two independent experiments consisting of a total of 16 animals per RNAi group. (**D**) Gene expression levels 15 dpfi to determine RNAi efficiency for single and triple RNAi of *Smed-PARP* genes. Interestingly, RNAi of *Smed-PARP-1* and *-2*, resulted in an increase in *Smed-PARP-3* gene expression. (**E**) Graph depicts gene expression of markers specific to neoblasts and their post-mitotic progeny (i.e., *Smed-Piwi-1*, *Smed-Prog-1*, and *Smed-AGAT-1* genes are represented via color coding, respectively) for animals 15-days into the phenotype. (**D**,**E**) All gene expression values are relative to the internal control clone H.55.12e. RNA extractions consisted of greater than 10 animals per group. (**F**) Putative GO term enrichment derived from PlanNET predicts the Smed protein function based off of the human protein interactome [36]. It is predicted that *Smed-PARP-1*, *-2*, and *-3* have a conserved function in regulating DNA dependent, ADP-Ribosylation, and protein modification biological processes. (**G**) Heatmap representing DNA damage marker gene expression levels for *Smed-Ku70* and *Smed-Rad51* 15 dpfi. Expression levels are as follows: low (blue), high (red) and relative to control (pink). (**H**,**I**) Quantification and visual representation of increased DNA damage levels determined by RAD51 protein levels 30-days post triple RNAi of *Smed-PARP-1*, *-2*, and *-3*. Increase in RAD51 expression was determined by the intensity of the signal relative to the animal surface area, using ImageJ software. All graphs represent mean ± SEM Statistics were obtained by two-way ANOVA; ns: no significance, * < 0.05, **  < 0.001, ***  < 0.0005, and ****  < 0.0001. Scale bar is 200 µm.

To determine the role of DNA dependent PARylation during tissue homeostasis and cellular turnover, we performed RNA-interference (RNAi) by microinjecting animals with double-stranded RNA (dsRNA). Planarians were injected with dsRNA for each *Smed-PARP* gene five times over 30 days (30 dpfi; Figure 3A). Under this RNAi schedule, no macroscopic or behavioral abnormalities were observed in intact animals except for a reduction in surface area in animals subjected to *Smed-PARP-1(RNAi)* (Supplementary Figure S4A). Immunostaining with an anti-phosphorylated histone 3 (H3P) antibody (labels dividing cells) and evaluation of cell death with the TUNEL assay revealed both cell division and cell death remained at similar levels between the control (injected with GFP-dsRNA) and the experimental groups 30 dpfi (Figure 3B,C). These results suggest that cellular turnover in planarians does not depend on DNA dependent PARylation.

To determine if the lack of a phenotype post RNAi was due to compensatory effects of the three DNA-dependent PARPs, we performed simultaneous downregulation with double and triple RNAi. Surprisingly, we did not observe, after 30 days, any morphological or cellular defects in the experimental group. Moreover, we determined that by 15 dpfi the levels of gene expression were severely downregulated. This finding strongly supports the efficiency of the RNAi strategy and suggests downregulation of each individual PARP gene may affect the expression levels of the other PARP genes (i.e., *Smed-PARP-3*; Figure 3D). Furthermore, we were able to determine that both individual or simultaneous downregulation of the PARP genes by 15 dpfi was able to alter the expression of neoblasts and the post-mitotic progeny markers (*Smed-Piwi-1*, *Smed-CyclinB*, and *Smed-Prog-1*, respectively) but did not change the levels of expression for the differentiated marker *Smed-AGAT-1* (Figure 3E and Supplementary Figure S4B). The results suggest that despite the stringent RNAi regimen of DNA-dependent *Smed-PARP* genes, there is no apparent effect on tissue homeostasis or tissue morphology in the intact animals despite alterations to gene expression found early in the phenotype.

To further investigate the putative function of PARP signaling in planarians, we first used the PlanNET database [36] to predict the planarian PARP protein-interaction network to that of the human. Based on this predictive assessment, gene ontology analysis suggested that DNA dependent and ADP ribosylation activities are among the most common and extensive putative biological processes in *Smed-PARP-1*, *-2*, and *-3* (Figure 3F and Supplementary Tables S1 and S2). To confirm the possible role of PARP signaling in DNA dependent functions, we performed qPCR using probes against genes critical for DNA repair in planarians (i.e., *Smed-Ku70* and *Smed-Rad51*) [18]. This analysis revealed that after 15 dpfi the expression of the DNA repair genes tended to decrease but when simultaneous RNAi was performed against the three PARP genes, the expression of *Smed-Ku70* and *Smed-Rad51* increased (Figure 3G). We expanded the analysis with immunostaining against the RAD51 protein, which is critical for the repair of DNA double-strand breaks [18,19,20] and found an important increase in the RAD51 signal after 30 dpfi of *Smed-PARP-1*, *-2*, and *-3* (Figure 3H,I). The results strongly supported the nuclear function and DNA repair of PARP signaling in planarians.

Lastly, to determine if the loss of DNA-dependent *Smed-PARP* expression results in alterations to the nervous tissue during tissue homeostasis, we evaluated gene expression of different markers associated with nervous tissue and the protein expression of VC-1 and SYNORF1 at 15 and 30 dpfi. Gene expression levels were for the most part consistent across individual *Smed-PARP-1*, *-2*, and *-3(RNAi)* at 15 dpfi except for the increase in expression for *collagen* and *TBH* that was more notorious after *Smed-PARP-2* (Supplementary Figure S5A). Likewise, there were no significant alterations to intact RNAi animal physiology of the photoreceptor, brain, and ventral nervous cords architecture (Supplementary Figure S5B–D). Collectively, the data suggest that PARP signaling plays a role in DNA repair with a minimal influence in tissue homeostasis.

### 2.4. Smed-PARP Genes Regulate Tissue Regeneration

**Figure 4:**
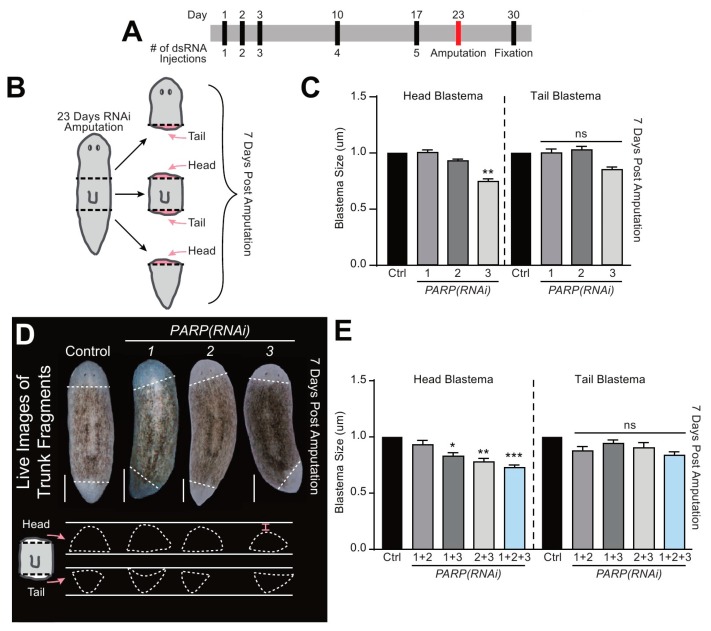
*Smed-PARP-3* is required for anterior-specific blastema formation. (**A**) Regeneration time course injection and amputation scheduled. (**B**) Graphic depicting sites of amputation. Animals were severed 23 dpfi, both above and below the pharynx resulting in the head, trunk, and regenerative tail fragments. (**C**) Measurements of the regenerative blastema area 7 dpa relative to the whole fragment area. (**D**) Representative live images of 7 dpa regenerative trunk fragments for the control and RNAi group. Below, are tracings of both anterior and posterior blastemas. Results show a significant decrease in anterior facing blastema areas of *Smed-PARP-3(RNAi)* animals (red bracket). (**E**) Blastema area for double and triple RNAi showing that *Smed-PARP-3(RNAi)* involvement stunts anterior blastema growth. (**C**–**E**) Data represents the pooling of fragments capable of regenerating head blastemas (i.e., anterior facing trunk fragment and tail fragment) and tail blastema are a pooling both the tail formation of the trunks and head fragments (reference Figure 4B). Single RNAi experiments were conducted in four independent biological replicates containing a total of 32 animals per RNAi group. As for the double and triple RNAi experiments, data represent two biological replicates resulting in a total of 16 individual amputations per condition. Graphs represent mean ± SEM of all the pooled head, trunk and tail fragments unless otherwise specified. Statistics were obtained by two-way ANOVA; ns: no significance, * < 0.05, **  < 0.001, and ***  < 0.0005. Scale bar is 200 µm.

Evidence suggests that PARylation plays a role in regeneration [11,37,38]. To discern its functional role in planarian tissue regeneration, we amputated animals along the anteroposterior axis and followed their repair process over seven days (Figure 4A,B). The three fragments generated upon amputation (i.e., head, trunk, and tail) regenerate either anterior or posterior blastemas depending on their location. For example, trunk fragments regenerate a head in their anterior facing wound, while the tail is regrown from the posterior facing wound (Figure 4B). To evaluate the process of repair, we measured the size of individual blastemas from either anterior or posterior facing wounds and plotted their size depending on the orientation of the blastema. The differential pigmentation between pre-existing tissue and the newly created blastema was used to determine the size of the regenerated tissue. The counts were obtained from control and animals subjected to the individual and simultaneous *Smed-PARP(RNAi)*. In the case of single RNAi, control and animals subjected to *Smed-PARP-1*, and *-2(RNAi)* formed both anterior and posterior blastemas with similar sizes. However, the group subjected to *Smed-PARP-3(RNAi)* showed a reduction of about 25% in the size of the anterior but not the posterior blastemas (*n* = 32/32; Figure 4C,D and Supplementary Figure S6). Furthermore, animals subjected to double and triple RNAi, using our optimized multiple RNAi strategy [18,19,39,40,41], we found that in any combination subjected to *Smed-PARP-3(RNAi)* there was a consistent reduction in the size of the anterior-facing head blastemas (*n* = 16/16; Figure 4E and Supplementary Figure S7). We also analyzed blastema formation in each regenerating fragment individually (i.e., head, trunk, and tail) and found that head blastema was always compromised in fragments obtained from *Smed-PARP-3(RNAi)* (Supplementary Figure S7). Taken together, the results suggest *Smed-PARP-3* is required for proper scaling of regenerating anterior blastemas.

### 2.5. Smed-PARP-3 Regulates Cell Death and Neurogenesis during Regeneration

**Figure 5:**
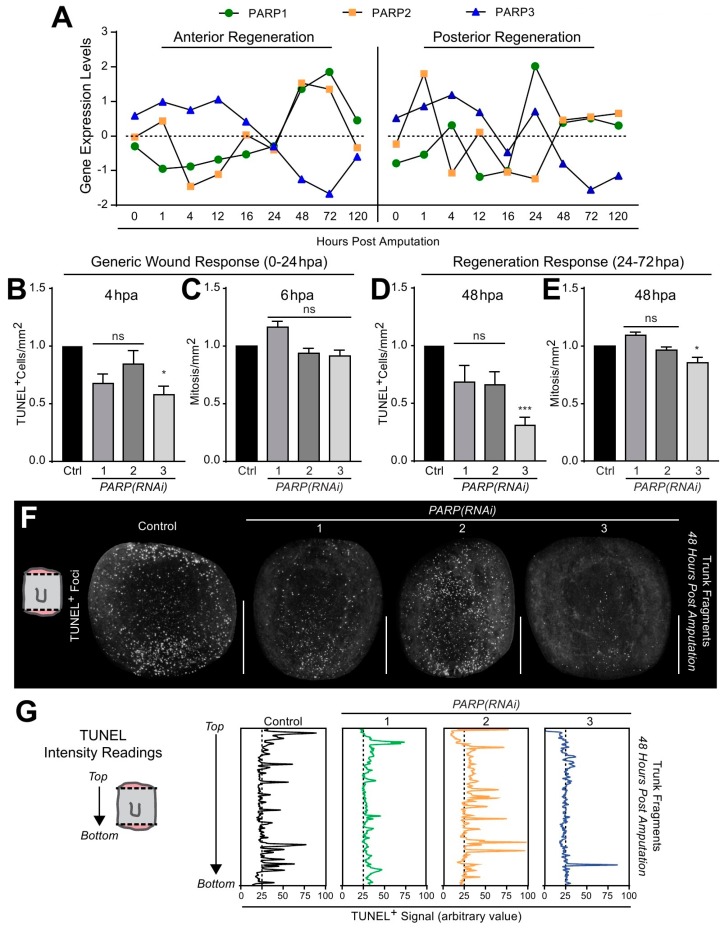
*Smed-PARP-3(RNAi)* alters cell death patterns during planarian regeneration. (**A**) Gene expression levels of *Smed-PARP-1*, *-2*, and *-3* (i.e., green, orange, and blue, respectively). Data is derived from RNA sequencing conducted during anterior or posterior regeneration time course [47]. Notice that *Smed-PARP-3* expression is elevated during the first 24 hpa known as the generic wound response. (**B**) TUNEL positive foci quantified 4 hpa, where a localized cell death response is established at the wound site. (**C**) Quantification of the system-wide mitotic burst 6 hpa showing no significant change in events. (**D**) The graph represents cell death within the regenerative response 48 hpa, reveling a significant decline in the system-wide death response in *Smed-PARP-3(RNAi)* animals. (**E**) Results of mitotic events during the localized wave of proliferation seen at 48 hpa. (**F**) Representative images of cell death within regenerating trunk fragments 48 hpa. TUNEL positive cells found system-wide in the regenerating trunk fragment of the control group; however, *Smed-PARP-3(RNAi)* animals seem to have a posterior-specific accumulation of cell death. (**G**) Intensity readings of TUNEL positive foci in regenerating trunks 48 hpa depicting the biased cell death response found in *Smed-PARP-3(RNAi)* animals. RNAi experiments were conducted in three independent biological replicates containing a total of 24 animals per RNAi group. Graphs represent mean ± SEM of all the pooled head, trunk and tail fragments unless otherwise specified. Statistics were obtained by two-way ANOVA; ns: no significance, * < 0.05, ** < 0.001, *** < 0.0005, and **** < 0.0001. Scale bar 200 µm.

Tissue regeneration requires a highly tuned regulation between cell death and cellular proliferation [42]. In planarians, the initial generic wound response, bioelectric signaling, and mechanical tissue contraction drive wound closure [43,44,45,46,47]. Injury-driven cell death and cellular proliferation occur stereotypically within the first few hours [42,44]. Recently, we have identified that regeneration requires the activation of the DNA damage response (DDR) [20]. Specifically, we showed that *Smed-PARP-3* expression peaks between 0 and 3 hours post-amputation (hpa) and it gradually declines over time, which coincides with an increase in expression 6–12 hpa of other DDR proteins including *Smed-PARP-1* and *-2* [20]. Extending the analysis during regeneration of anterior and posterior tissues throughout the first 120 hpa [47], demonstrates a similar trend, whereby *Smed-PARP-3* expression is elevated in both anterior and posterior wound types during the first 12–16 hpa, and it gradually decreases over time as *Smed-PARP-1* and *-2* increase between 24 and 72 hpa (Figure 5A).

The generic wound response in planarians takes place within the first 24 hpa, and the regenerative response between 24 and 72 hpa follows this. The generic wound response is characterized by an increase in localized apoptosis near the wound-site at 4 hpa, followed by a systemic neoblast proliferation-response occurring around 6 hpa [44,45]. We found that in *Smed-PARP-3(RNAi)* animals, there was a significant decrease in cell death at 4 hpa, while the systemic mitotic response was similar for control and experimental groups during the generic wound response (Figure 5B,C and Supplementary Figure S8A,B). However, within the regeneration response (48 hpa), we observed a substantial reduction for both cell death and cellular division in animals subjected to *Smed-PARP-3(RNAi)* (Figure 5D,E and Supplementary Figure S8C,D). Interestingly, despite the massive decline in apoptotic events throughout the trunk fragments, we identified that the spatial distribution of apoptotic cells in *Smed-PARP-3(RNAi)* animals were restricted to the posterior facing wound and severely reduced or absent in anterior facing wounds (*n* = 24/24; Figure 5F,G and Supplementary Figure S9). Furthermore, the individual assessment of regenerating heads, trunks, and tails evidenced that anterior facing wounds in tail fragments lacked cell death events unlike the posterior facing wounds of the head and trunks fragments that showed reduced and limited system-wide apoptotic events (Figure 5F,G and Supplementary Figure S9). Together, these results suggest that *Smed-PARP-3(RNAi)* may alter the spatial distribution of cell death in anterior facing wounds.

**Figure 6:**
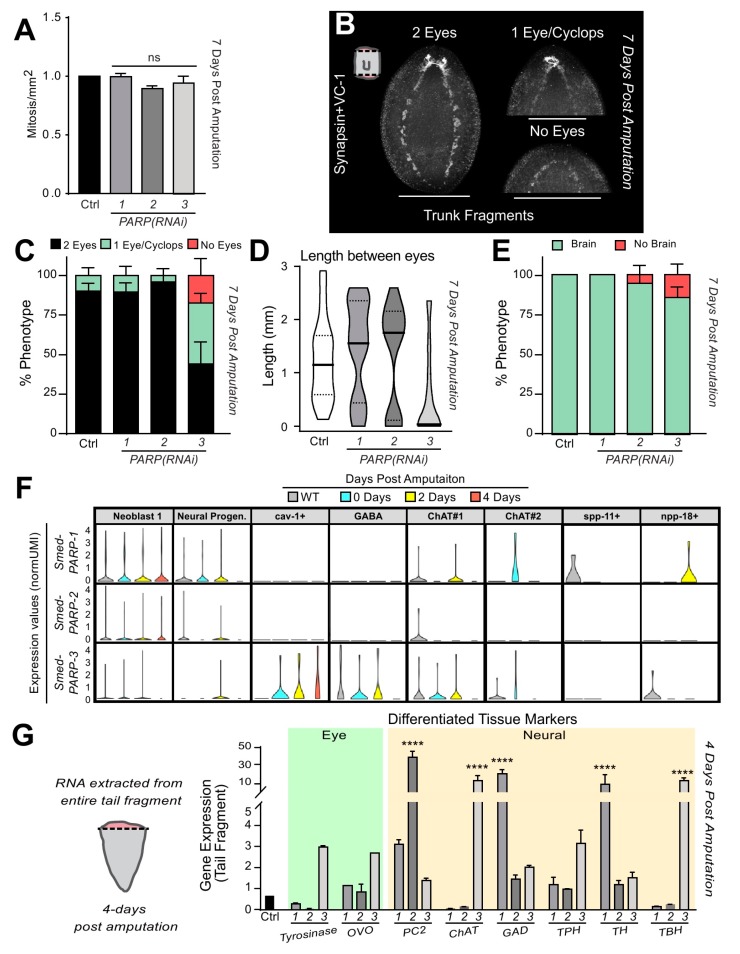
*Smed-PARP-3* expression is required for neural differentiation during regeneration. (**A**) Quantification of mitotic events seven-days post amputation show no significant alterations for regenerating *Smed-PARPs* relative to the control. (**B**) Seven-day regenerating trunk fragments stained with antibodies specific for planarian brain/ventral nerve cords and eye cup pigmentation (i.e., SYNORF1 and VC-1, respectively). Images are representative of the three categories used to quantify PARylation effect on differentiation during regeneration (e.g., two eyes, one eye or Cyclops, and no eyes). (**C**) Quantification of the percent of animals exhibiting a specific eye phenotype. (**D**) Violin plots depicting the distributing of the length between the two eye pigments 7 dpa. The average lengths: 1.25 ± 0.75 mm, 1.44 ± 0.87 mm, and 1.34 ± 0.95 mm with *Smed-PARP-3(RNAi)* animals were containing the smallest mean distance of 0.56 ± 0.79 mm. (**E**) Percent of the animals containing brain deformities 7 dpa. (**F**) Expression values (normUM) of *Smed-PARP-1*, *-2*, and *-3* during a regeneration time course for the neural lineage tree derived from the single-cell transcriptome planarian atlas [49]. Notice that expression levels for *Smed-PARP-3* are elevated in the neural lineages specific to *Cav-1+*, *GABA*, and *ChAT#1*, required for proper central nervous system development. (**G**) qPCR analysis of gene expression from four-day regenerating tail fragments (e.g., anterior regeneration). Expression levels for differentiated tissues targeting eye tissues (i.e., *Smed-OVO* and *Smed-Tyrosinase*) and central nervous system/neural peptides (i.e., *Smed-PC2*, *-ChAT*(cholinergic), *-GAD*(GABAergic), *-TPH*(Dopaminergic), *-TH*(Serotonergic), and *-TBH*(Octopaminergic)) were assessed for *Smed-PARP-1*, *-2*, and *-3(RNAi)* regenerating tail fragments. Gene expression values are relative to the internal control clone H.55.12e. RNA extractions consisted of greater than 10 animals per group. (**B**–**D**) Neural phenotype experiments are an average of three biologically independent experiments resulting in a pool of 24 amputated animals per group. Graphs represent mean ± SEM of the pooled trunk and tail fragments unless otherwise specified. Scale bar is 200 µm.

Next, we asked whether morphogenetic defects accompanied the reduced blastema size and deficient cell death in the anterior facing wounds. Despite no changes in mitotic activity at 7 dpa (Figure 6A), regeneration was still impaired. This led us to stain 7-day regenerating fragments with antibodies specific for the neural eye receptors/cups and brain tissues (e.g., anti-VC-1 and anti-SYNORF1, respectively). First, we looked at photoreceptor pigmentation, which is evident in the anterior blastema at 7 dpa (*n* = 24 per RNAi group). The results demonstrate that control and *Smed-PARP-1*, and *-2(RNAi)* animals displayed an average of 10% of animals containing phenotypes of one eye or a Cyclops (e.g., 13% ± 8%, 10% ± 8%, and 5% ± 6%, respectively; Figure 6B,C). On the other hand, animals subjected to *Smed-PARP-3(RNAi)* had over 50% animals with defective eye pigmentation including three times more with one eye/a Cyclops (e.g., 39% ± 10%) and about 35% ± 10% with no eye pigmentation (Figure 6B,C). Furthermore, measurements between the eyecups stained by VC-1 [48] revealed a reduction in length between the eyes for *Smed-PARP-3(RNAi)* animals when compared to the control and *Smed-PARP-1*, and *-2(RNAi)* animals, (i.e., 0.56 ± 0.79 mm, 1.25 ± 0.75 mm, 1.44 ± 0.87 mm, and 1.34 ± 0.95 mm; Figure 6D). We also identified that about 15% of *Smed-PARP-3(RNAi)* regenerating fragments had scarce or no brain tissue (Figure 6B,E). Moreover, we wanted to determine if loss of PARP during tail regeneration would affect posterior neural formation thus, we analyzed both the length of the ventral nervous cords and the distance between their connection in the tail and found no significant change relative to the control 7 dpa (Supplementary Figure S10A,B). These findings prompted us to re-evaluate single-cell expression analysis of *Smed-PARP* genes during specific time points of regeneration. The analysis identified that *Smed-PARP-*3 is largely expressed within various neuron types including cholinergic and GABAergic neurons that are required for the proper function of the nervous system (Figure 6F and Supplementary Figure S11A–E) [49]. To test the possibility that *Smed-PARP* genes regulate neural expression 4 dpa, we performed qPCR focusing on genes marking differentiated tissues of the eyes and nervous system within RNA extracted from 4 dpa regenerating tail fragments (e.g., regenerating anterior tissue; Figure 6G). We found that 4 dpa loss of *Smed-PARP-1*, *-2*, and *-3(RNAi)* altered neural gene expression patterns in regenerating tail fragments (i.e., *Smed-PC2*, *-ChAT*, *-GAD*, *-TH,* and *-TBH*). Moreover, *Smed-PARP-3(RNAi)* increased expression levels of *Smed-ChAT* and *Smed-TBH* (markers of cholinergic and octopaminergic neurons), which is consistent with the in silico data. We did notice an increase in gene expression for eye markers (i.e., *Smed-Tyrosinase* and *-OVO*) in *Smed-PARP-3(RNAi)* regenerating fragments but the values were not significant relative to the control. Together, these results suggest that DNA-dependent *Smed-PARP* genes are crucial for neural expression patterns and differentiation during the regeneration process.

## 3. Discussion

We demonstrated that PARP signaling regulated large-scale tissue regeneration in planarians. Specifically, dysfunctional *Smed-PARP-3* led to anterior specific impairment of both injury-induced cell death and nervous tissue regeneration. Our findings evidenced evolutionary conservation of three PARP homologs with a DNA-dependent and ADP-ribosylation putative functions. The putative GO terms analysis also suggests functions associated with cellular differentiation, cell division, and apoptosis appear conserved in planarians. Together, the biological process found in planarians is consistent with similar functions of PARylation in various invertebrate and vertebrate organisms [1,10,23].

The gene expression analysis demonstrated that PARP genes were ubiquitously expressed in *S. mediterranea*. These data evidenced that while *Smed-PARP-1* and *-2* expression was mostly enriched in stem cells, *Smed-PARP-3*, was largely transcribed in post-mitotic cells; including neural progenitors and neuronal clusters. The reasons for the differential expression were not readily evident, but it is possible that PARP signaling in planarians may involve functions associated with DNA repair and DNA damage independent roles. Indeed, we confirmed PARP-related DNA repair functions appeared conserved in planarians, which is consistent with recent findings [20]. Planarians constantly renew adult tissues and display astonishing reparative capacity upon injury, which depends on adult stem cells and differentiated tissues that guide them. Thus, finding the expression of *Smed-PARP* genes in stem cells and differentiated cells offer simplified grounds, to resolve in vivo, the interactions between PARP signaling and the genetic network regulating their function across different cell types in the adult body. Moreover, the evolutionary conservation of PARP signaling, together with a large amount of stem cells in planarians represents an exciting paradigm to learn about the role of PARP signaling in adult stem cells. This is relevant because the presence of PARP in self-renewing cells represent a promising opportunity to enhance cancer therapy in different types of tumors in the colon, lung, head and neck, and cervix [50,51,52,53,54].

The results suggest that DNA dependent PARP signaling in planarians was more relevant for tissue regeneration than in the context of cellular turnover. Additional experiments are needed to rule out whether cellular effects such as cell division, migration, or differentiation are affected in long term tissue renewal. The homeostatic effects of PARylation may be overshadowed by the dominant suppression of alternative enjoining through the core DNA repair signaling pathways (i.e., nonhomologous end joining and homologous recombination) [55,56,57]. Nonetheless, the finding that *Smed-PARP-3* is a regulator of regeneration is consistent with our previous work, where we found its expression sharply activated within the first three hours post-injury [20] and recent findings by the Aboobaker group [22]. The mechanistic effect of *Smed-PARP-3* activation early during the generic wound response is unknown, but it is possible that PTM may facilitate the timely and spatial location of cell death near the anterior facing wound. In planarians, PTM involving phosphorylation, ubiquitination, and chromatin remodeling are widely present and influence cellular response to injury [12]. PTM through PARylation may also involve repression of cell cycle progression to emplace DNA repair, as shown in other experimental models [58,59,60]. Furthermore, PARP-3 has been shown to interact independently of the other DNA-dependent PARPs thus, this may explain the exacerbated effect seen upon triple RNAi as these genes may present a compensatory role of the DNA-dependent PARPs [20]. In this regard, we propose *Smed-PARP-3* has conserved roles in regulating apoptosis, but it remains unclear whether neural defects observed in *Smed-PARP-3(RNAi)* regenerating animals are due to defective apoptosis of neural progenitors and/or a deficient differentiation that compromise regeneration of the nervous system in the anterior part of the animal.

The findings presented here introduce planarians as a tractable model to study PARP signaling in the context of adult stem cell fueled tissue renewal and regeneration. Future studies will address how and when *Smed-PARP* homologs engage in DNA repair and/or independent genomic stability functions. Further analysis would be needed to resolve the compensatory roles of *Smed-PARP* homologs and their regulation of regeneration in the nervous system.

## 4. Material and Methods

### 4.1. Planarian Culture

Seven-day starved *Schmidtea mediterranea* from the asexual clonal strain CIW4 were used in all experiments; maintenance as previously described [61].

### 4.2. Protein Identification and Expression Analysis

Identification of PARylation family members in the planarian model was conducted by BLASTing the 17 human PARP members into the planarian PlanMine3.0 database [24]. Subject IDs were obtained for *Smed-PARP-1*, *-2*, and *-3* (e.g., dd-Smed_v6_10338_0_1, dd-Smed_v6_6154_0_8, and dd-Smed_v6_2611_0_1, respectively). Percent identities and protein conservation analysis were determined by Clustal Omega and NCBI Conserved domains (https://www.ncbi.nlm.nih.gov/Structure/cdd/wrpsb.cgi). Phylogenetic bootstrap consensus tree of PARP-1, -2, and -3 across an array of species was created using MEGA7 software. Protein domain models were created through IBS1.03 (GPS; http://ibs.biocuckoo.org/download.php). For expression analysis of *Smed-PARP* homologs, current single-cell RNA-sequencing databases were used (e.g., Digiworm, Shiny, and Planosphere) [26,34,35,49].

### 4.3. RNA Interference and Regeneration Experiments 

Starving planarians were subjected to five single and pooled synthesized dsRNA microinjections over thirty days. dsRNA was synthesized as previously described [62]. Synthesis for PARPs 1–3 required the following primer sets: *Smed-PARP-1*: forward—CGGTGGCCGATTGTATG GTA and reverse—GGTCCTGGTAAAGGTGAGCC, *Smed-PARP-2*: forward—GGATTGTTGAGTG AGGGGCA and reverse—ATGTGGATTGGTCAGGAGCG, and *Smed-PARP-3*: forward—CCATGC CACAAGAGTTTGCG and reverse—CCAAGCAAAAGGCTGACCTG. As for regeneration experiments, planarians received five microinjections of dsRNA and were subjected to head, trunk, and tail amputation seven days before the completion of the 30-day injection time course. Animals were monitored and fixed at various time points throughout the seven days. In all cases (i.e., blastema, H3P, and TUNEL staining) the data in the main figures represents a pool of all three fragments (i.e., head, trunk, and tail fragments) from three independent biological replicates, unless otherwise noted. Each replicate consisted of 8 worms thus producing 24 fragments. In total 24 animals were amputated resulting in 72 fragments per timepoint and stain. The supplemental images represent the pooled data per fragment that was used to produce the main pool of data in the main figure.

### 4.4. Immunohistochemistry

Animals were fixed at various time points using Carnoy’s solution [18,19,41]. Specimens were subjected to blocking for 4 h and then incubated in primary antibody overnight: α-H3P 1:250 (Millipore Cat# 05-817R); α-VC1 1:10,000 [48](Kind gift of K. Watanabe); SYNORF1 1:100 (Developmental Studies Hybridoma Bank); and RAD51 1:500 (Abcam Cat#ab13847). After a series of 7 × 1 h washes, animals were blocked and incubated in the secondary antibody overnight: Alexa488 (1:400) goat anti-mouse (Invitrogen Cat# 673781), goat-anti-mouse HRP IgG 1:1000 (Life Technologies), and Alexa568 (1:800) goat anti-rabbit (Invitrogen Cat# 11036).

### 4.5. TUNEL Assay

Animals subjected to TUNEL assay were fixed using 10% NAC as previously described [18,41]. The TUNEL assay ApopTag Red in situ Apoptosis Detection Kit was used for all experiments (TUNEL Kit; Millipore, Cat# S7165). Tagging of animals was followed as previously described [18,41], and animals were mounted and immediately imaged.

### 4.6. Quantitative RT-PCR

Gene expression analysis post RNAi is relative to the ubiquitously expressed clone H.55.12e [18,19,30,41]. Values corresponded to the mean of triplicated samples derived from an RNA pool extracted from > 10 animals each. Fold change represents standardized expression levels of RNAi/Control.

### 4.7. Whole Mount In-Situ Hybridization (WISH)

The whole mount in-situ hybridization (WISH) protocol was based on previously published work [63].

### 4.8. Imaging and Data Processing

Images were obtained using a Nikon AZ-100 multi-zoom microscope and NIS Elements AR 3.2 software. Brightness and contrast were adjusted with Adobe Photoshop. Furthermore, surface area measurements and foci counts were calculated with ImageJ software (https://imagej.nih.gov/ij/). Foci-specific images (i.e., TUNEL or H3P) were counted and normalized to the area (mm^2^) using ImageJ. As for neural characterization (e.g., photoreceptors, brain, and ventral nerve cords) the length or area measuring tool was used to quantify these parameters. Photoreceptor and ventral cord analysis measured length between structures (e.g., between eyes and ventral cord connotation) and length of the ventral cords began from the bottom of the brain lobes to the connection interface between the left and right ventral cord. In all cases, normalization was used to compare across biological replicates.

### 4.9. Statistical Analysis

Data are expressed as the fold change of the mean ± standard error of the mean (SEM). One-way or two-way ANOVA statistics were performed in Prism, GraphPad Software Inc.

## Supplementary Materials

Supplementary materials can be found at https://www.mdpi.com/1422-0067/21/3/875/s1.
