# Supplementary material for: Poly(ADP-Ribose) Polymerase-3 Regulates Regeneration in Planarians
Source: Int J Mol Sci. 2020 Jan 29;21(3):875. doi: 10.3390/ijms21030875 (PMC7038108; doi:10.3390/ijms21030875)
Supplement: Supplementary file 1 [file ijms-21-00875-s001.pdf]

Supplementary Materials

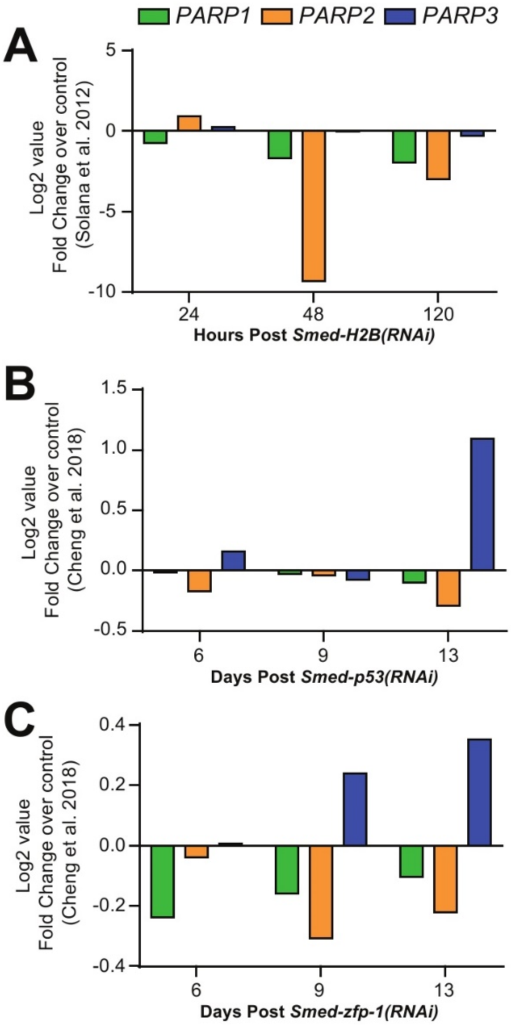

Figure S1. *Smed-PARP-1* and *-2* are expression levels are altered by neoblast depletion. (**A**) Log2 values of expression after *Smed-H2B(RNAi)* depletion over a 120-h time course. (**B**–**C**) *Smed-PARP-1*, *-2 and -3* expression levels over a 13-day time course post-RNAi of *Smed-p53* and *Smed-zfp-1*; key regulators of neoblast function. In all graphs, *Smed-PARP-1*, *-2* and *-3* are depicted by the following colors: green, orange and blue, respectively. Data points were derived from [25,31,32].

A

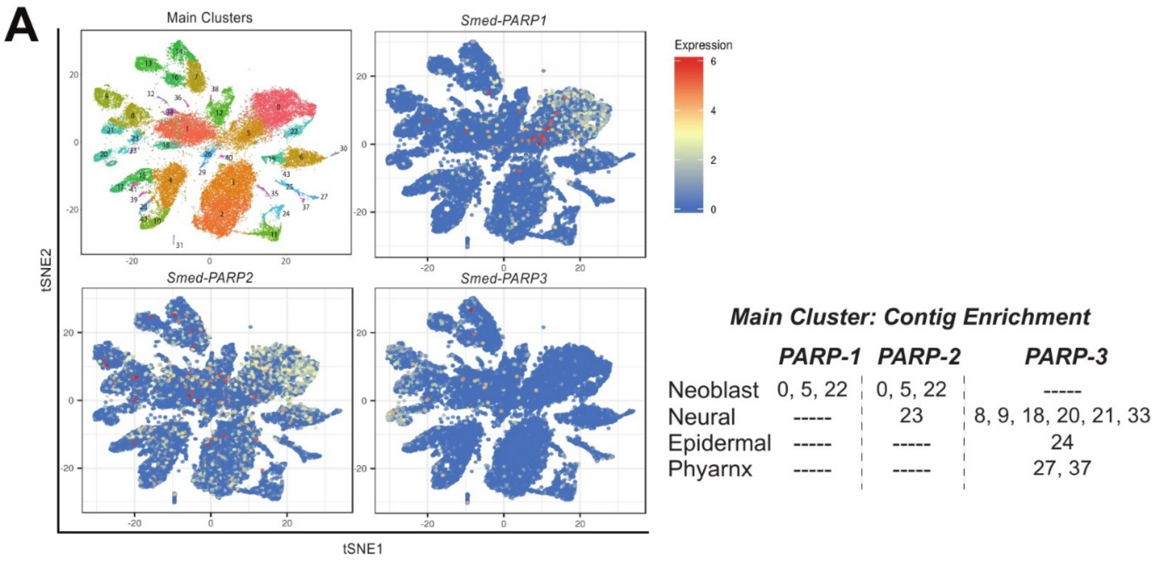

Main Cluster: Contig Enrichment

	PARP-1	*PARP-2*	*PARP-3*
Neoblast	0, 5, 22	0, 5, 22	-----
Neural	-----	23	8, 9, 18, 20, 21, 33
Epidermal	-----	-----	24
Phyarnx	-----	-----	27, 37

B

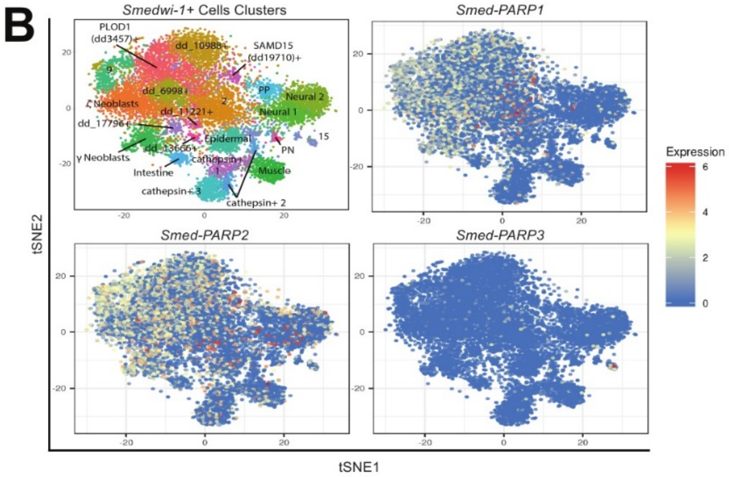

C

Sub-Cluster: Contig Enrichment

	PARP-1	*PARP-2*	*PARP-3*
Cathepsin+	0	3	4
Epidermal	2	2	5, 11
Smedwi+1	1, 18	4	15
Muscle	-----	5	-----
Neural	-----	-----	5, 20, 21, 26, 30, 31, 41, 53
Parenchymal	-----	-----	5
Pharynx	-----	-----	6

Figure S2. tSNE plots depicting *Smed-PARP-1*, *-2*, and *-3* within planarian cell types. (**A**) tSNE expression plots are derived from single-cell RNA sequencing analysis. Low expression levels are seen in blue, while is mild expression and red is high expression levels. The graph on the top left shows the 42 major cluster types. *Smed-PARP-1* and *-2* expression levels are found to be high throughout the neoblast clusters while the expression of *Smed-PARP-3* is restricted to the neural clusters (see table to the right). (**B**) The reference *Smedwi-1*+ cell cluster tSNE plot can be located on the top left corner. Isolating expression levels of *Smed-PARPs* in the *Smedwi-1*+ cell clusters revel that *Smed-PARP-1* and *-2* are found within the neoblast populations. Moreover, *Smed-PARP-3* expression is almost void, except within *Smedwi-1*+ cluster number 15. (**C**) Sub-clusters contig enrichment table. Data derived from digiworm database [34].

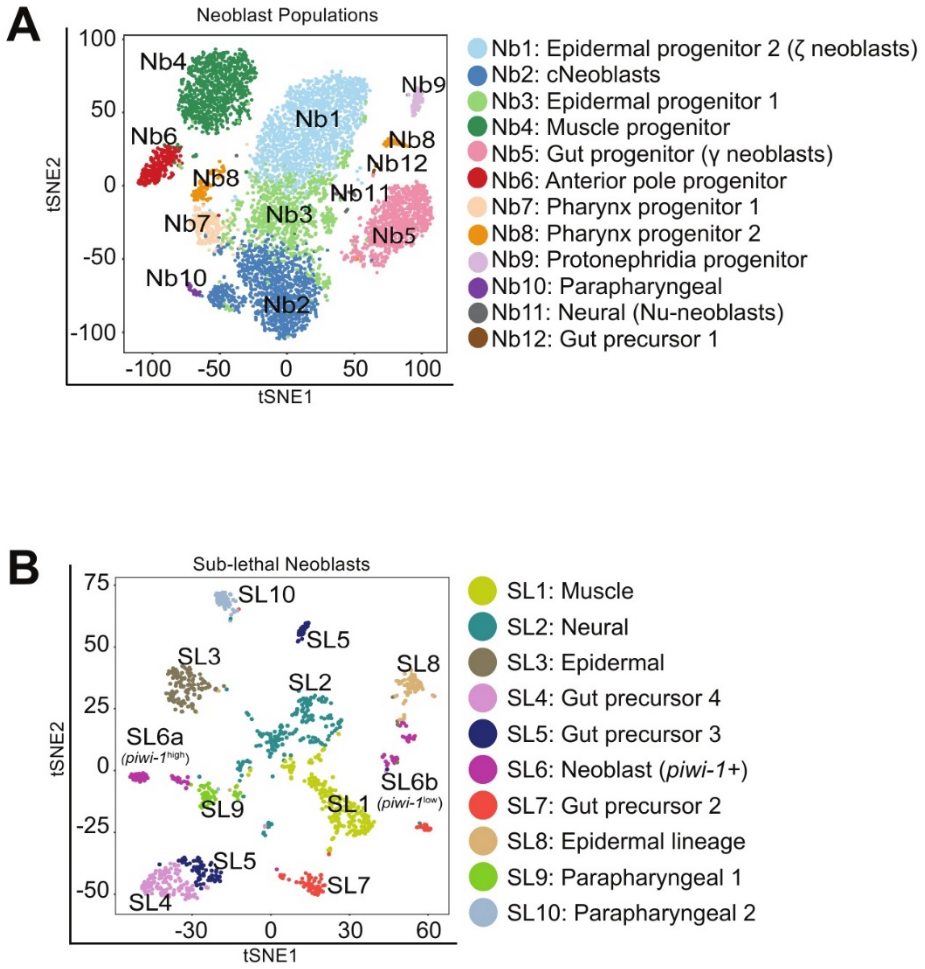

Figure S3. Neoblast and Sub-lethal neoblast population legend. (**A**,**B**) tSNE expression plots (neoblast and sub-lethal neoblst populations, respectively) are derived from single-cell RNA sequencing analysis accessed from Planosphere fate mapping atlas [35]. This image corresponds to Figure 2E.

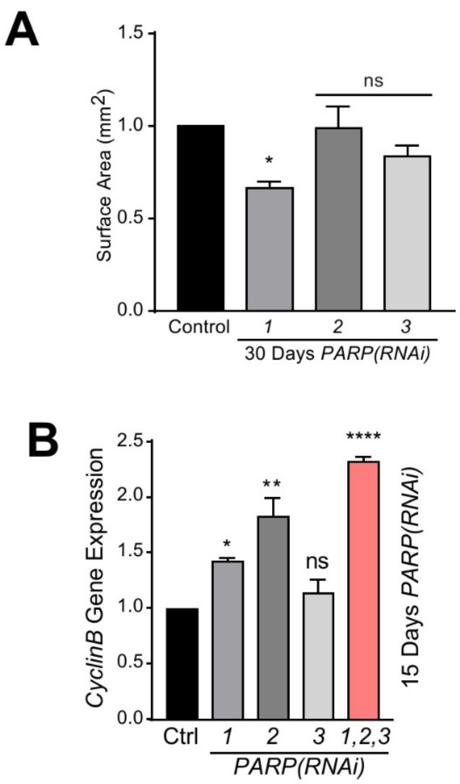

Figure S4. Surface area measurements and *CyclinB* expression levels upon loss of *Smed-PARPs*. (**A**) Surface area per mm^2 of animals 30 dpfi shows a significant reduction in *Smed-PARP-1(RNAi)* animal size relative to the control. (**B**) Gene expression levels of *CyclinB* 15-days into the phenotype. Gene expression values are relative to the internal control clone H.55.12e. RNA extractions consisted of greater than 10 animals per group. All graphs represent mean ± SEM Statistics were obtained by two-way ANOVA; ns: no significance, * < 0.05, ** < 0.001, and **** < 0.0001.

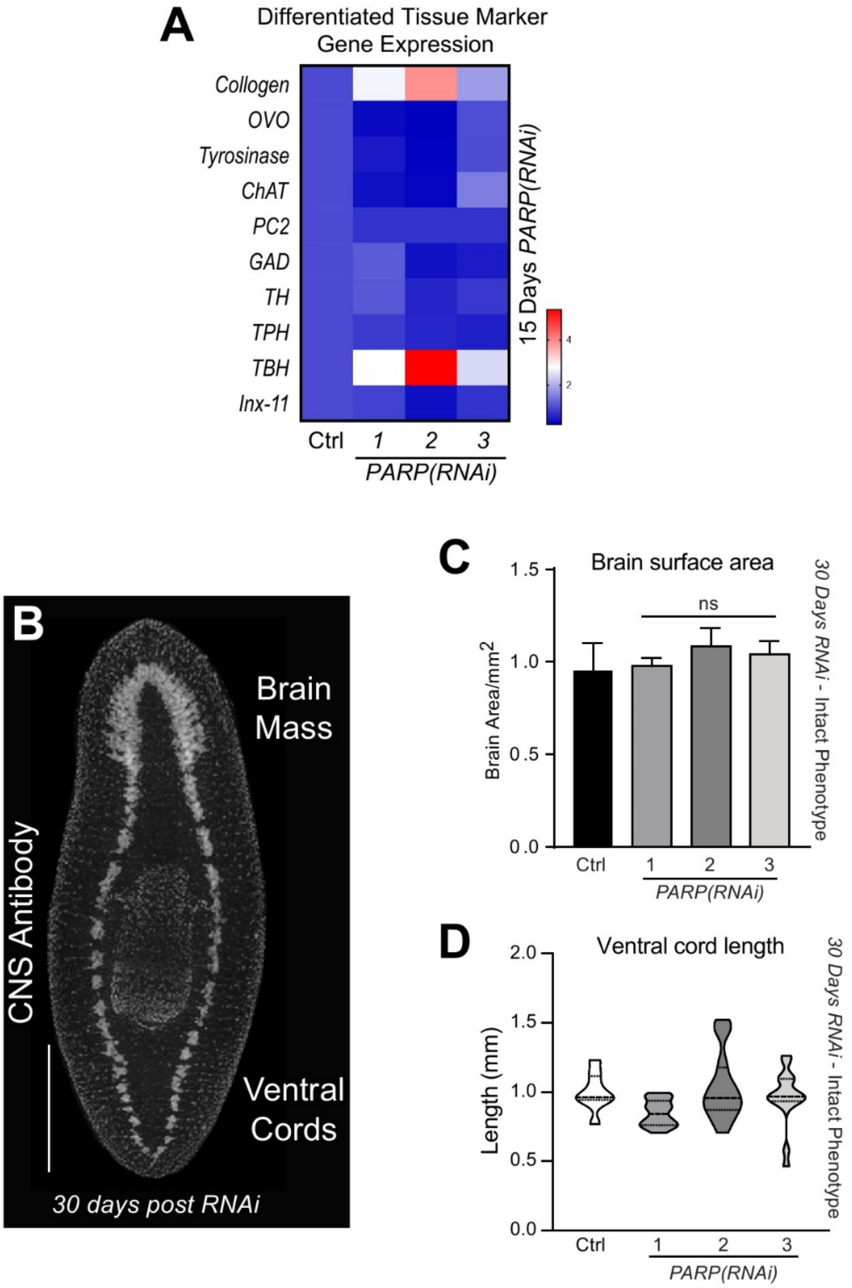

Figure S5. Loss of *Smed-PARPs* do not alter neural differentiated tissues during tissue homeostasis. (**A**) Heatmap representing gene expression levels, 15 dpfi, of markers for differentiated tissues targeting muscle (i.e., *Smed-Collagen*), eye tissues (i.e., *Smed-OVO* and *Smed-Tyrosinase*) and central nervous system/neural peptides (i.e., *Smed-ChAT*, *-PC2*, *-GAD*(GABAergic), *-TH*(Serotonergic), *-TPH*(Dopaminergic), *-TBH*(Octopaminergic) and *-Inx-11*). Expression levels are as follows: low (dark blue), high (red) and relative to control (purple). Gene expression values are relative to the internal control clone H.55.12e. RNA extractions consisted of greater than 10 animals per group. (**B**) Whole mount immunostaining against anti-SYNORF1 specific for planarian brain/ventral neve cords. This image is a representative of the control and RNAi groups. (**C**–**D**) Quantification of brain area and ventral neural cord length post 30-day RNAi of *Smed-PARP-1*, *-2*, and *-3* results in no alterations to brain surface area. Graph graphs represent mean ± SEM Statistics were obtained by two-way ANOVA; ns: no significance.

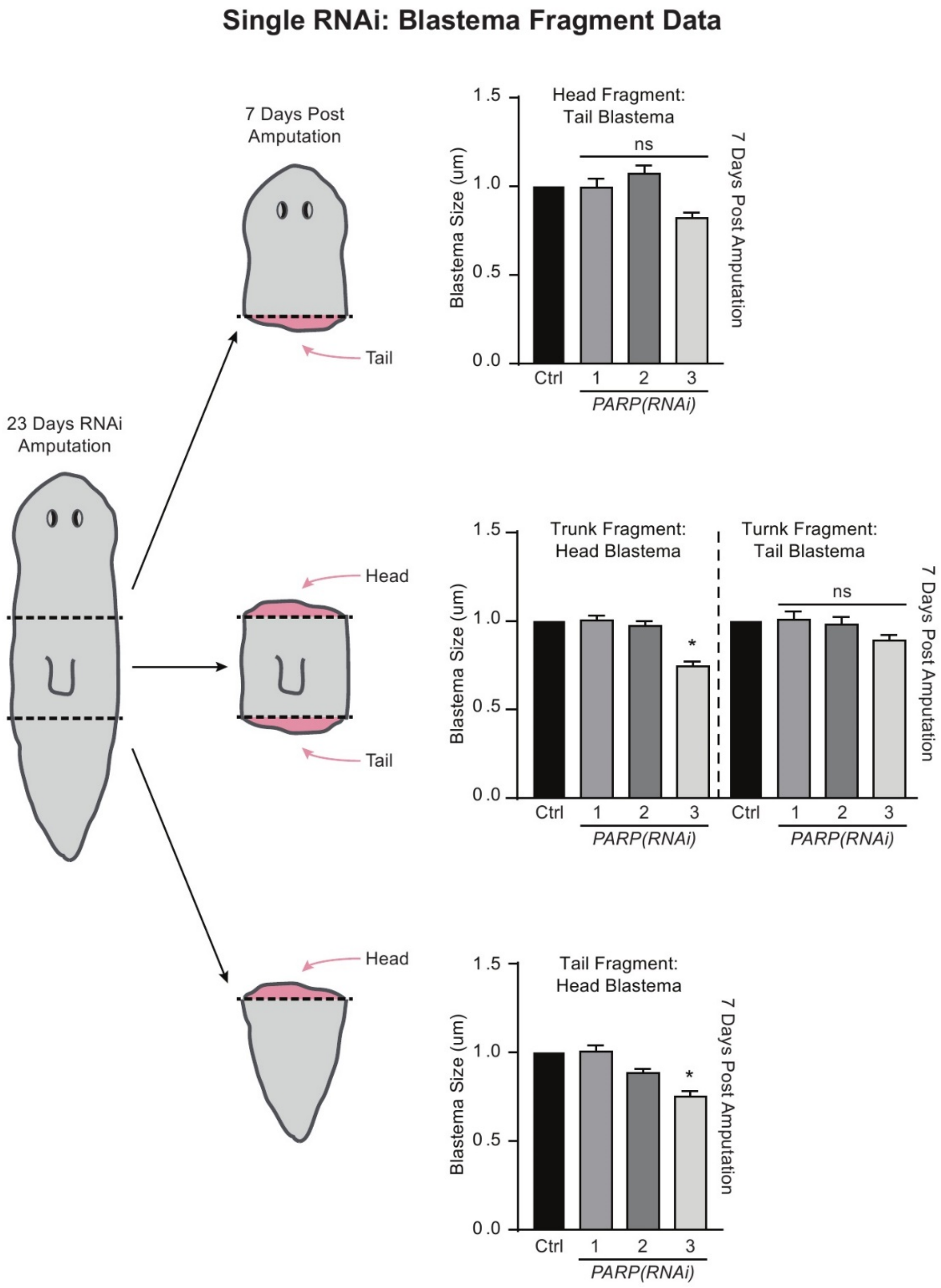

Figure S6. Blastema size per fragment in single RNAi groups. Graphs depict the non-pooled blastema size per head, trunk, and tail fragment 7 dpa. These results were pooled to generate the table in Figure 3G. Single RNAi experiments were conducted in four independent biological replicates containing a total of 32 animals per RNAi group. Graphs represent mean ± SEM Statistics were obtained by two-way ANOVA; ns: no significance, * < 0.05.

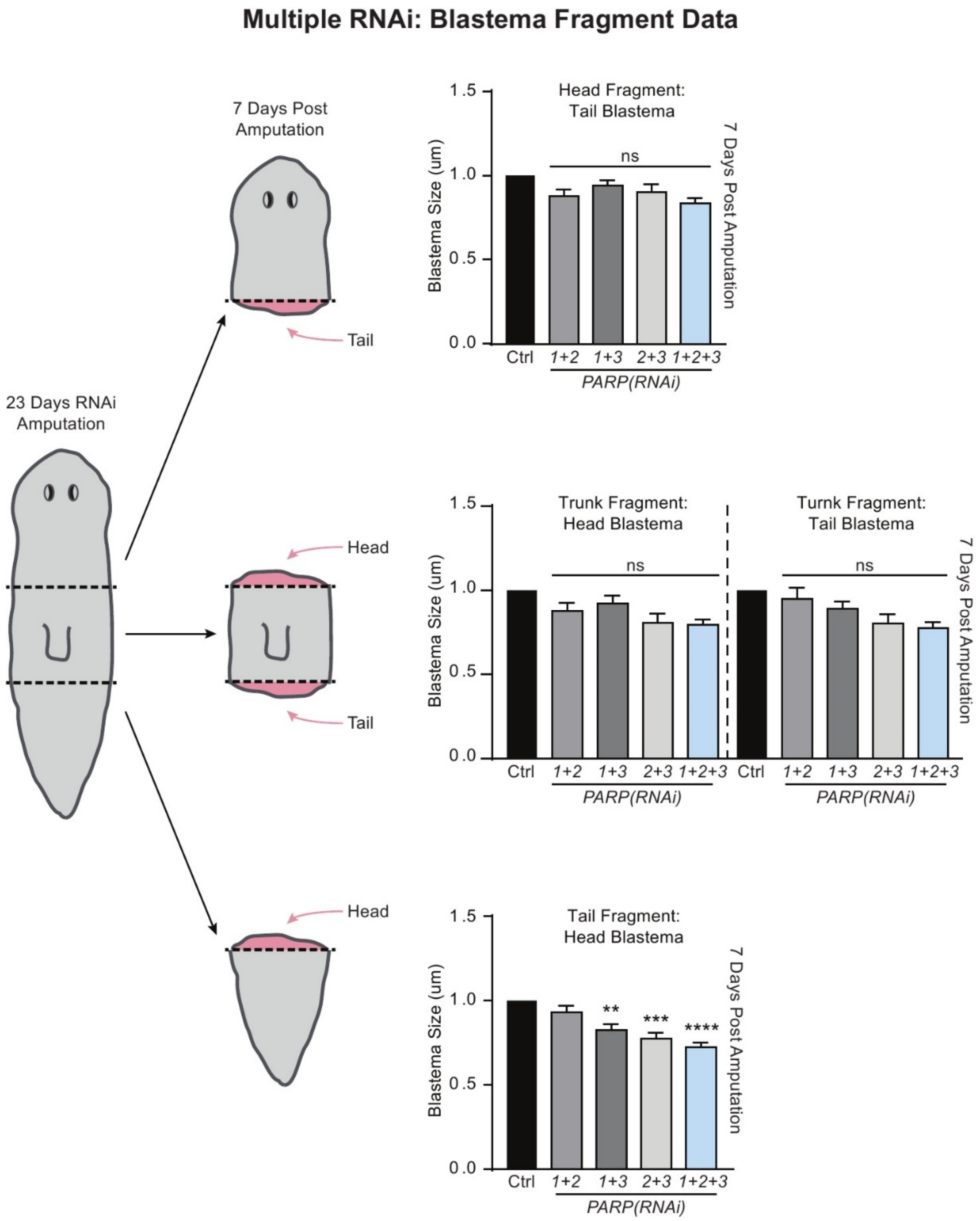

Figure S7. Blastema size per fragment in double and triple RNAi groups. Graphs depict the non-pooled blastema size per head, trunk, and tail fragment 7dpa of the multiple RNAi groups. These results were pooled to generate the table in Figure 3I. Double and triple RNAi experiments, data represent two biological replicates resulting in a total of 16 individual amputations per condition. Graphs represent mean ± SEM Statistics were obtained by two-way ANOVA; ns: no significance, ** < 0.001, *** < 0.0005, and **** < 0.0001.

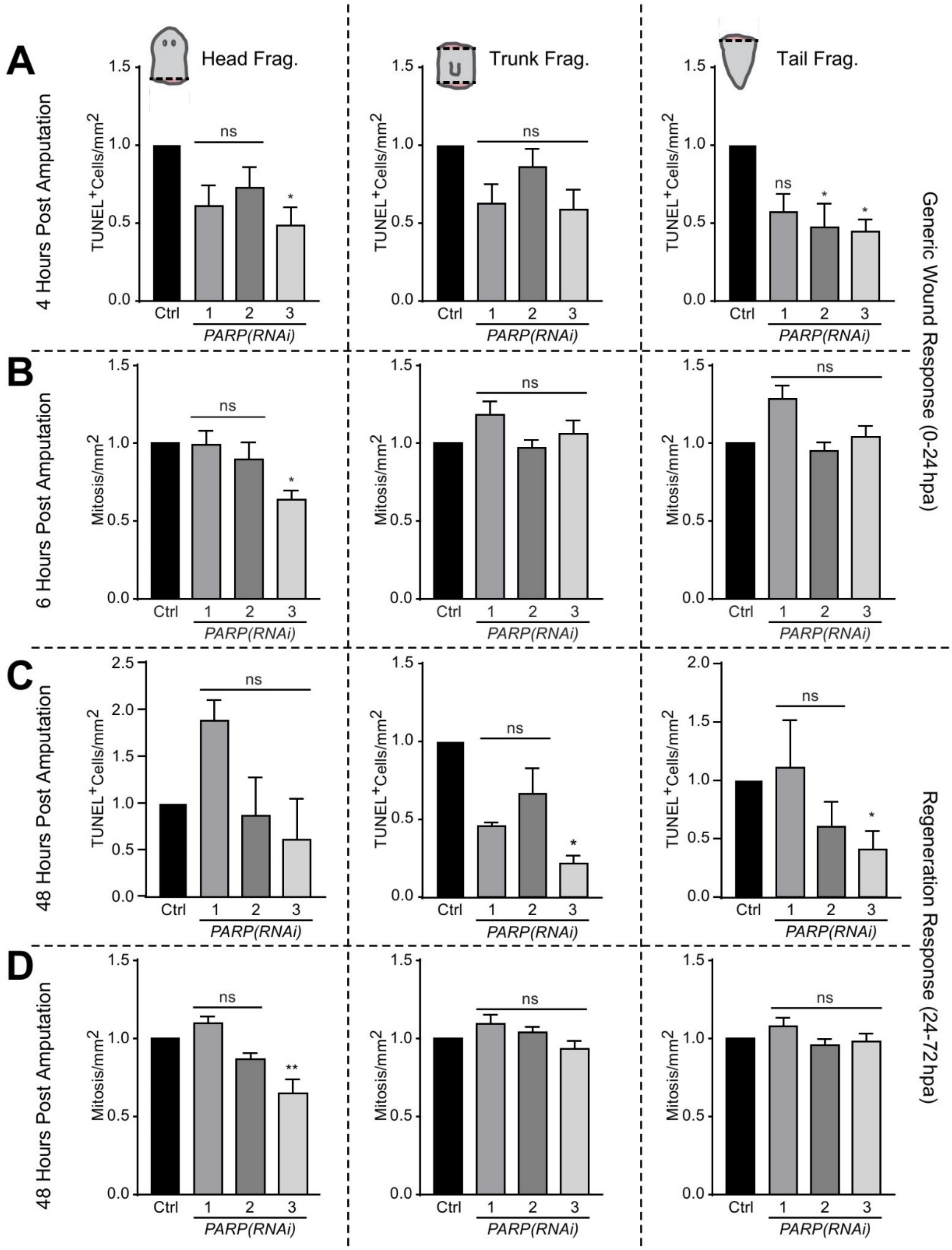

Figure S8. Quantification of cell death and mitosis per regenerating fragment. All graphs depict the non-pooled TUNEL or H3P positive foci (e.g., cell death and cell division, respectively) at various timepoints post-regeneration per fragment (e.g., head, trunk and tail). Data were pooled to create graphs in Figure 4B–E. (**A**) The quantification of cell death 4 hpa per fragment. (**B**) The mitotic response quantified at 6 hpa. (**C**) TUNEL positive foci quantification 48 hpa. (**D**) Quantification of mitotic events 48 hpa per regenerating fragment. Graphs represent mean ± SEM Statistics were obtained by two-way ANOVA; ns: no significance, * < 0.05, ** < 0.001.

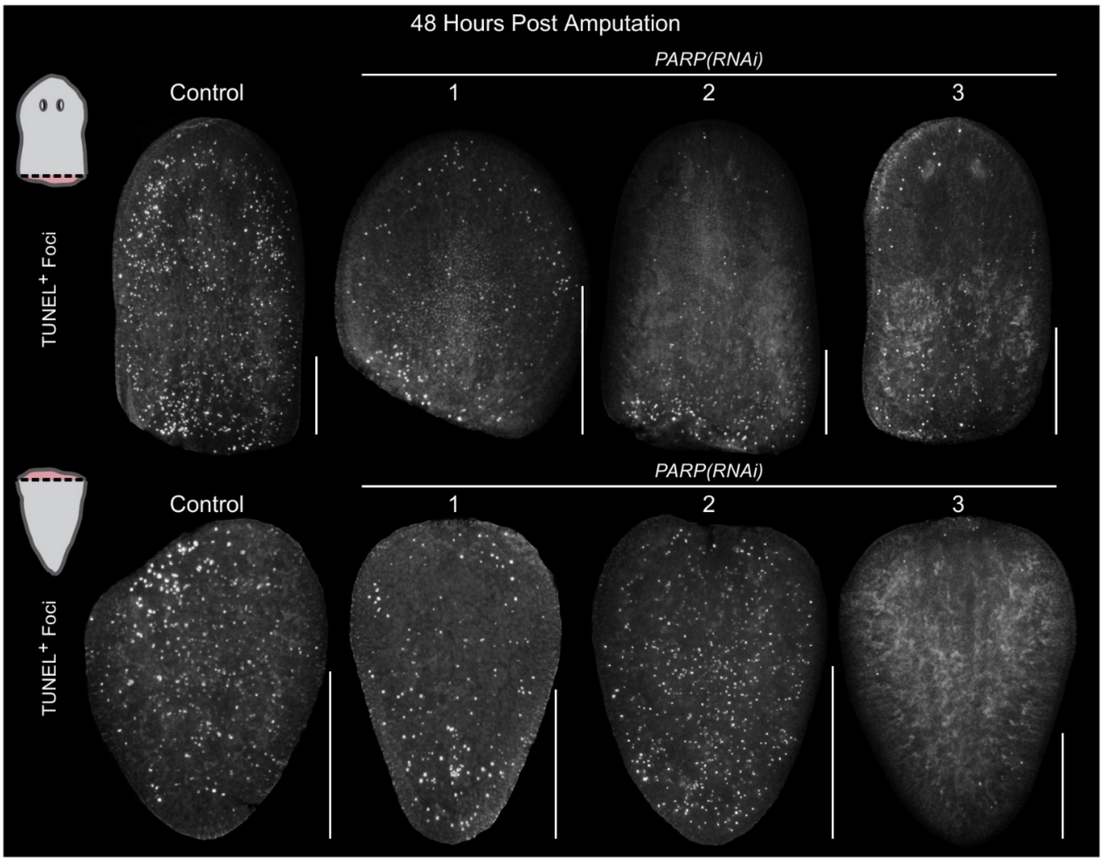

Figure S9. Cell death is lost in anterior facing wounds upon *Smed-PARP-3(RNAi)*. Representative images at 48 hpa of regenerating heads and tail fragments (top and bottom panel, respectively). Fragments show a distinct spread of cell death within the control and *Smed-PARP-1* and *-2(RNAi)* animals. Interestingly, *Smed-PARP-3(RNAi)* head fragments did contain a reduced system-wide cell death response, unlike the regenerating tail fragments that had little to no cell death present. Scale bar 200 μm.

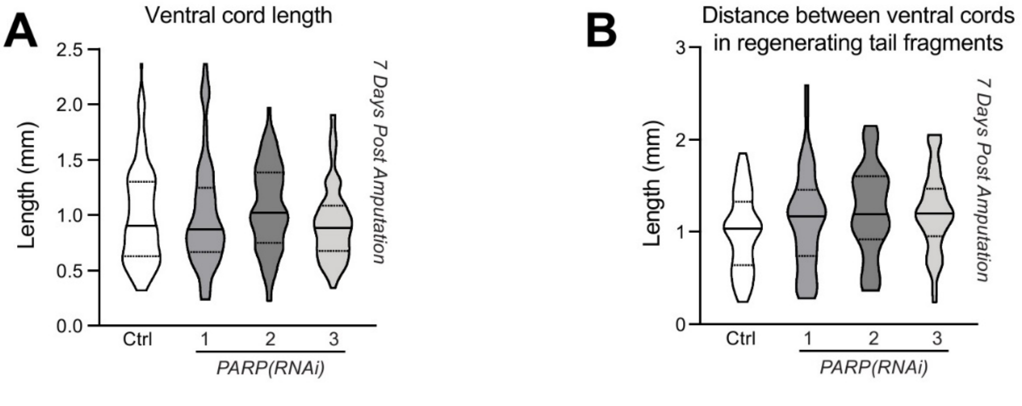

Figure S10. *Smed-PARP-1*, *-2*, and *-3(RNAi)* does not alter neural tissue morphology during tail-specific regeneration. (**A**,**B**) Violin-plots analyzing the ventral nervous cord length and distance between the left and right cord 7 dpa of the control and of *Smed-PARP-1*, *-2*, and *-3*.

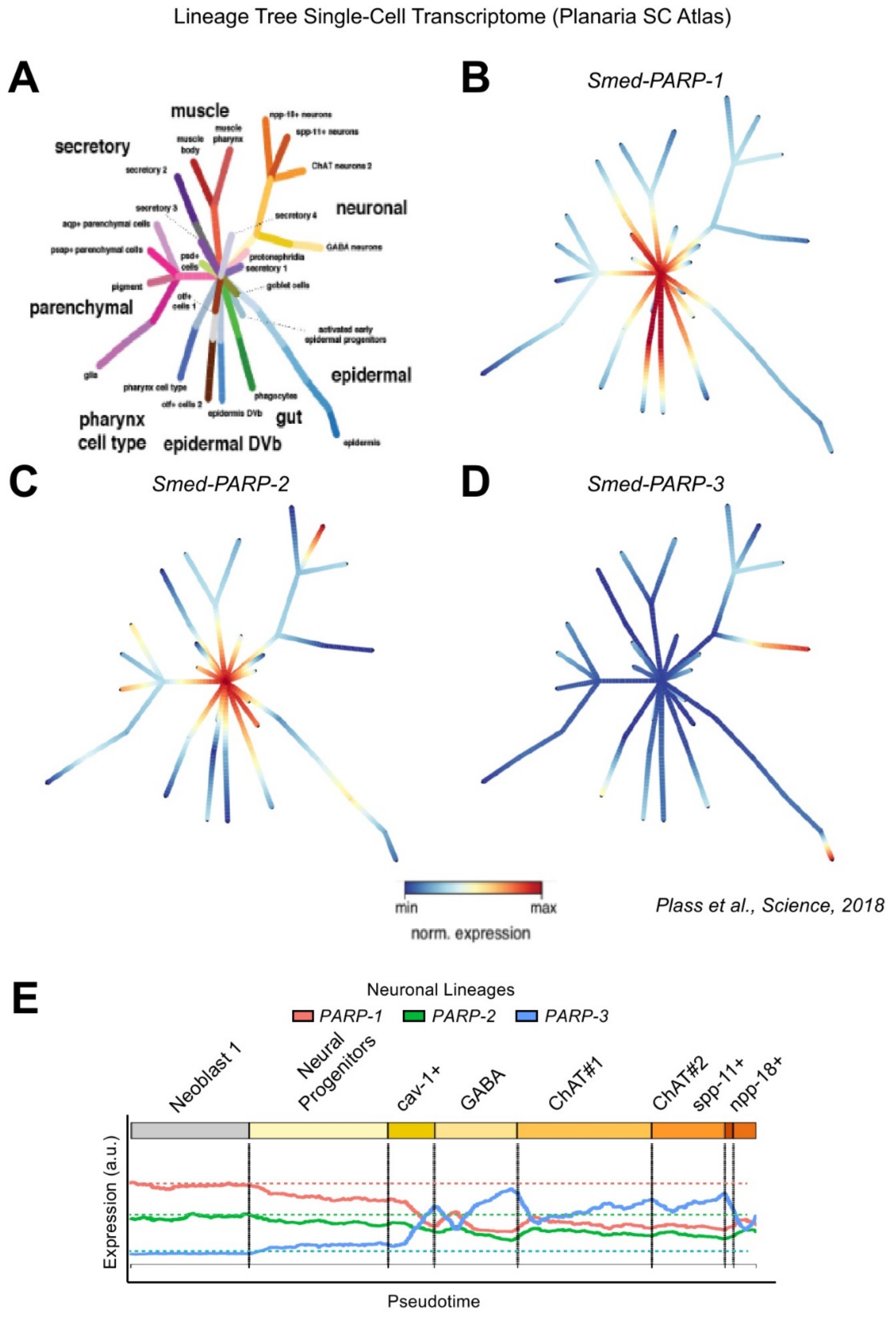

Figure S11. The expression patterns of *Smed-PARP-1*, *-2*, and *-3* confirm neural specificity of *Smed-PARP-3*. (**A**–**D**) Linage maps of *Smed-PARP-1*, *-2*, and *-3* expression patterns within the different stem-cell linages of intact non-regenerative planarian derive from Plass et al., 2018 [49]. Expression levels confirm that *Smed-PARP-3* is highly expressed within the nervous tissues during tissue homeostasis. (**E**) Pseudotime expression tracking of the neuronal lineages reveals that *Smed-PARP-3* expression is found to be elevated in that *Cav-1+*, *GABA ChAT#1/#2*, *spp-11+*, and *npp-18+* neural cell lineages.

GO Term: Biological Process	
**PARP1**	
GO:0060391	Positive Regulation Of Smad Protein Import Into Nucleus
GO:0042769	DNA Damage Response, Detection Of DNA Damage
GO:0023019	Signal Transduction Involved In Regulation Of Gene Expression
GO:0016540	Protein Autoprocessing
GO:2000679	Positive Regulation Of Transcription Regulatory Region DNA Binding
GO:0045944	Positive Regulation Of Transcription From RNA Polymerase Ii Promoter
GO:0006302	Double-Strand Break Repair
GO:0006471	Protein ADP-Ribosylation
GO:0006351	Transcription, DNA-Templated
GO:0000122	Negative Regulation Of Transcription From RNA Polymerase Ii Promoter
GO:0006289	Nucleotide-Excision Repair
GO:0006974	Cellular Response To DNA Damage Stimulus
GO:0006281	DNA Repair
GO:0006366	Transcription From RNA Polymerase Ii Promoter
GO:0000724	Double-Strand Break Repair Via Homologous Recombination
GO:0006367	Transcription Initiation From RNA Polymerase Ii Promoter
GO:0051103	DNA Ligation Involved In DNA Repair
GO:0044267	Cellular Protein Metabolic Process
GO:0006284	Base-Excision Repair
GO:0010467	Gene Expression
GO:0016925	Protein Sumoylation
GO:0007179	Transforming Growth Factor Beta Receptor Signaling Pathway
GO:0034599	Cellular Response To Oxidative Stress
GO:0032869	Cellular Response To Insulin Stimulus
GO:0007005	Mitochondrion Organization
GO:0043687	Post-Translational Protein Modification
GO:1903827	Regulation Of Cellular Protein Localization
GO:0033683	Nucleotide-Excision Repair, DNA Incision
GO:0010613	Positive Regulation Of Cardiac Muscle Hypertrophy
GO:0070212	Protein Poly-ADP-Ribosylation
GO:0030225	Macrophage Differentiation
GO:0070911	Global Genome Nucleotide-Excision Repair
GO:0000715	Nucleotide-Excision Repair, DNA Damage Recognition
GO:0006273	Lagging Strand Elongation
GO:0032042	Mitochondrial DNA Metabolic Process
GO:0036211	Protein Modification Process
GO:0043504	Mitochondrial DNA Repair
**PARP2**	
GO:0097191	Extrinsic Apoptotic Signaling Pathway
GO:0006284	Base-Excision Repair
GO:0006471	Protein ADP-Ribosylation
GO:0006281	DNA Repair
GO:0051103	DNA Ligation Involved In DNA Repair
GO:0006273	Lagging Strand Elongation
**PARP3**	
GO:0006281	DNA Repair
GO:0000723	Telomere Maintenance
GO:0051103	DNA Ligation Involved In DNA Repair
GO:0006273	Lagging Strand Elongation
GO:0051106	Positive Regulation Of DNA Ligation
GO:0060236	Regulation Of Mitotic Spindle Organization
GO:1990166	Protein Localization To Site Of Double-Strand Break
GO:0006302	Double-Strand Break Repair
GO:0006471	Protein ADP-Ribosylation

Table S1. Predicted Gene Ontology terms for biological processes for DNA-dependent Smed-PARPs. Putative GO term enrichment derived from PlanNET predicts the Smed protein function based off of the human protein interactome [36]. The list of predicted biological processes that Smed-PARPs -1, -2 and -3 are involved in. Table supports Figure 3F.

GO Term: Cellular Component	
**PARP1**	
GO:0005634	Nucleus
GO:0005654	Nucleoplasm
GO:0005730	Nucleolus
GO:0005739	Mitochondrion
GO:0016020	Membrane
GO:0005667	Transcription Factor Complex
GO:0043234	Protein Complex
GO:0000784	Nuclear Chromosome, Telomeric Region
GO:0005635	Nuclear Envelope
**PARP2**	
GO:0005634	Nucleus
GO:0005737	Cytoplasm
GO:0005654	Nucleoplasm
GO:0005730	Nucleolus
**PARP3**	
GO:0005634	Nucleus
GO:0005737	Cytoplasm
GO:0005814	Centriole
GO:0035861	Site Of Double-Strand Break

GO Term: Molecular Function	
**PARP1**	
GO:0070412	R-Smad Binding
GO:0042826	Histone Deacetylase Binding
GO:0003677	DNA Binding
GO:0008270	Zinc Ion Binding
GO:0003950	NAD+ ADP-Ribosyltransferase Activity
GO:0008134	Transcription Factor Binding
GO:0051287	NAD Binding
GO:0005515	Protein Binding
GO:0044822	Poly(A) RNA Binding
GO:0019901	Protein Kinase Binding
GO:0003910	DNA Ligase (ATP) Activity
GO:0042802	Identical Protein Binding
GO:0019899	Enzyme Binding
GO:0047485	Protein N-Terminus Binding
	**PARP2**
GO:0003677	DNA Binding
GO:0003950	NAD+ ADP-Ribosyltransferase Activity
GO:0005515	Protein Binding
GO:0003910	DNA Ligase (ATP) Activity
	**PARP3**
GO:0003824	Catalytic Activity
GO:0003910	DNA Ligase (ATP) Activity
GO:0003950	NAD+ ADP-Ribosyltransferase Activity

Table S2. Predicted Gene Ontology terms for cellular components and molecular function for DNA-dependent Smed-PARPs. Putative GO term enrichment derived from PlanNET predicts the Smed protein function based off of the human protein interactome [36]. The list of predicted cellular components and molecular function that Smed-PARPs -1, -2 and -3 are involved in. Table supports Figure 3F.
